# Adaptive camouflage: what can be learned from the wetting behaviour of the tropical flat bugs *Dysodius lunatus* and *Dysodius* *magnus*

**DOI:** 10.1242/bio.026070

**Published:** 2017-07-27

**Authors:** Florian Hischen, Vladislav Reiswich, Desirée Kupsch, Ninon De Mecquenem, Michael Riedel, Markus Himmelsbach, Agnes Weth, Ernst Heiss, Oskar Armbruster, Johannes Heitz, Werner Baumgartner

**Affiliations:** 1 Institute of Biomedical Mechatronics, Johannes Kepler University of Linz, Altenbergerstr. 69, 4040 Linz, Austria; 2 Department of Cellular Neurobionics, Institute of Biology II, RWTH-Aachen University, Worringerweg 3, 52056 Aachen, Germany; 3 University of Bordeaux, Campus Talence, 351 Cours de la Libération, 33400 Talence, France; 4 Department of Botany II, University of Würzburg, Julius-von-Sachs-Platz 3, D - 97082 Würzburg, Germany; 5 Institute of Analytical Chemistry, Johannes Kepler University of Linz, Altenbergerstr. 69, 4040 Linz, Austria; 6 Tiroler Landesmuseum, Josef-Schraffl-Straße 2a, A-6020 Innsbruck, Austria; 7 Institute of Applied Physics, Johannes Kepler University of Linz, Altenbergerstr. 69, 4040 Linz, Austria

**Keywords:** Wetting, Bug biomimetics, Reflectance, Camouflage, Liquid-surface interaction, Laser structuring, Microstructures

## Abstract

The neotropical flat bug species *Dysodius lunatus* and *Dysodius magnus* show a fascinating camouflage principle, as their appearance renders the animal hardly visible on the bark of trees. However, when getting wet due to rain, bark changes its colour and gets darker. In order to keep the camouflage effect, it seems that some *Dysodius* species benefit from their ability to hold a water film on their cuticle and therefore change their optical properties when also wetted by water. This camouflage behaviour requires the insect to have a hydrophilic surface and passive surface structures which facilitate the liquid spreading. Here we show morphological and chemical characterisations of the surface, especially the cuticular waxes of *D. magnus*. Scanning electron microscopy revealed that the animal is covered with pillar-like microstructures which, in combination with a surprising chemical hydrophilicity of the cuticle waxes, render the bug almost superhydrophilic: water spreads immediately across the surface. We could theoretically model this behaviour assuming the effect of hemi-wicking (a state in which a droplet sits on a rough surface, partwise imbibing the structure around).  Additionally the principle was abstracted and a laser-patterned polymer surface, mimicking the structure and contact angle of *Dysodius* wax, shows exactly the behaviour of the natural role model – immediate spreading of water and the formation of a thin continuous water film changing optical properties of the surface.

## Abstract (precis)

**Summary:** The optical properties of some neotropic bark bugs change when wetted. The present work attempts to explain the phenomenon and transfer it to a polymer surface via laser structuring.

## INTRODUCTION

**Fig. 1.**
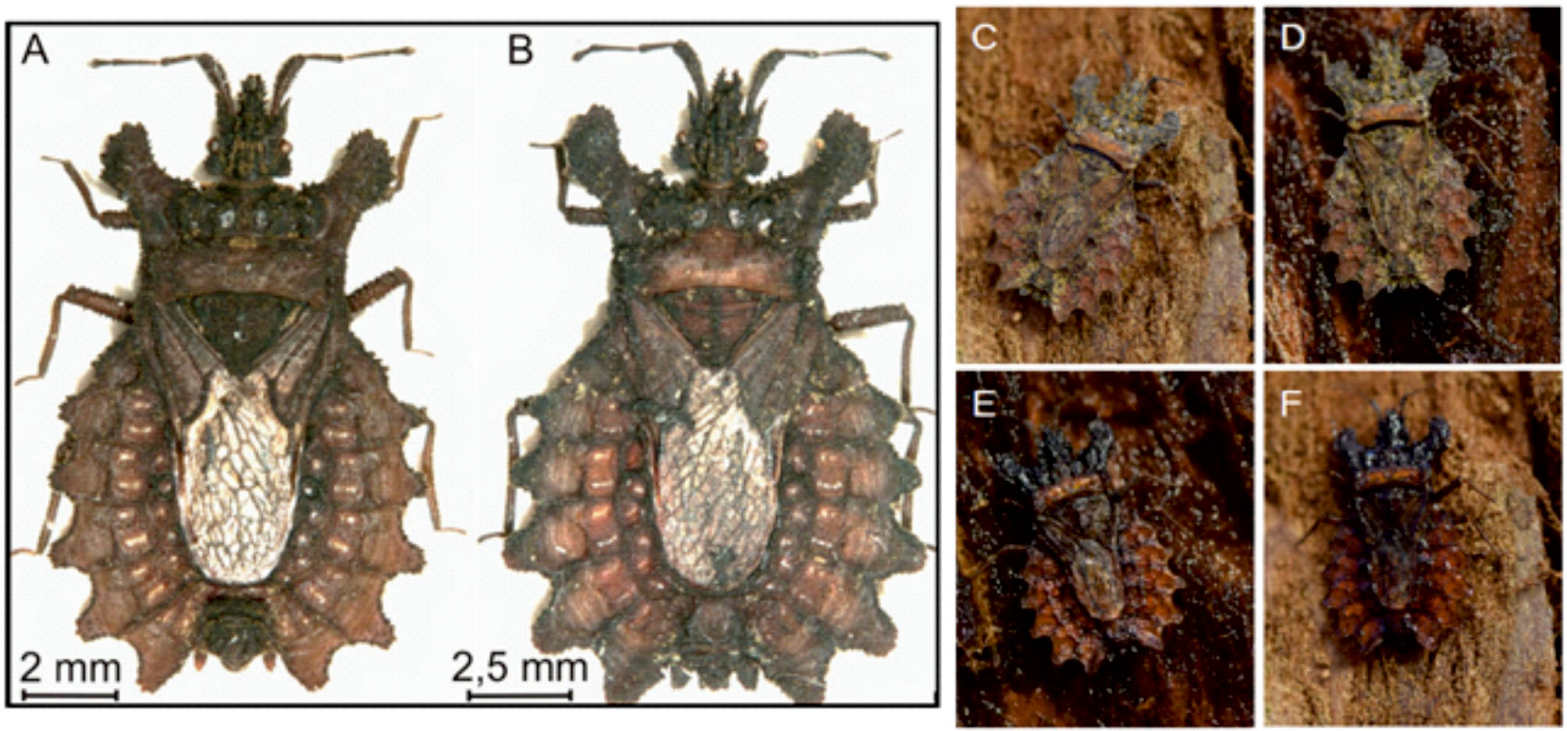
**Bark bugs and their camouflage on dry** **and wet bark.** A dry, adult specimen of *Dysodius lunatus* is depicted in (A) while a dry, adult specimen of *Dysodius magnus* is shown in (B). (C) Clearly, the camouflage of the dry animal on dry bark is excellent (in this case the bark of *Sequiadendron giganteum*). However, if the bark is wetted, the dry animal can be seen easily (D). (E) If, on the other hand, the animal is wetted itself, the colour changes to darker shades and the camouflage is excellent again. (F) The wet animal could of course easily be seen on dry bark. Images C-F were obtained at the Institute for Biomedical Mechatronics, JKU Linz, to reproduce the findings of Silberglied and Aiello (1980) in a better quality.

Flat bugs (Aradidae), also named bark bugs, comprise a family of insects in the order of Heteroptera (true bugs). In the temperate zone, these animals are often found on or under the loose bark of dead trees; while many tropical species are found in leaf litter or on fallen twigs and branches, or they dwell on bark. Most if not all members of the family are supposed to be mycophagous. Flat bugs are relatives of the more familiar shield and tree bugs (Pentatomidae/Scutelleridae) (Schuh and Slater, 1995). They typically rely on their excellent camouflage, i.e. their colouration, often mottled brown/black/grey/yellow, which makes them quite difficult for predators, like birds, to detect; this was, for example, investigated by Johansen et al. (2010). This study investigates the flat bug species *Dysodius lunatus* (Fabricius, 1794, Fig. 1A) and *Dysodius magnus* (Fig. 1B) (Heiss, 1990) which live on the outside of the bark of trees in the neotropics where they feed on fungi (Panizzi and Grazia, 2015). Their cryptic nature should be apparent from Fig. 1C where it is difficult to make out where the bark ends and the animal begins. However, these flat bugs face a problem: bark changes its appearance, namely its colour, if wetted. As can be observed in daily life, most tree bark becomes dark when wetted. This change of the albedo is due to total internal reflection within a liquid film on rough surfaces, as well as on a change of the reflection coefficient due to different refractive indices of water and the wetted material. Wetted bark is quite common in tropical rain forests, and as can be seen in Fig. 1D, a dry *Dysodius* specimen would be easily detectable on wet bark. Most terrestrial insects possess a highly hydrophobic cuticula (Holdgate, 1955), mainly due to waxes rendering these animals not wettable. For most insects, this water repellent behaviour is advantageous. This does not apply for the *Dysodius* species investigated here. Unlike most other insects, these flat bugs are easily wettable and change colour when wetted. This allows the animals to adapt to the moisture-induced colour change of the bark accordingly. This was first shown by Silberglied and Aiello (1980) and can be seen in Fig. 1C-F.

In the present study we tried to analyse this unusual wetting behaviour of insects. We characterised the epicuticular waxes chemically as well as morphologically and describe specialised wax glands of these animals. We describe the wetting behaviour theoretically and finally we succeed in reproducing the wetting behaviour of the insect's cuticle by using specially structured polymer foils.

## RESULTS

### Wetting of flat bugs

**Fig. 2.**
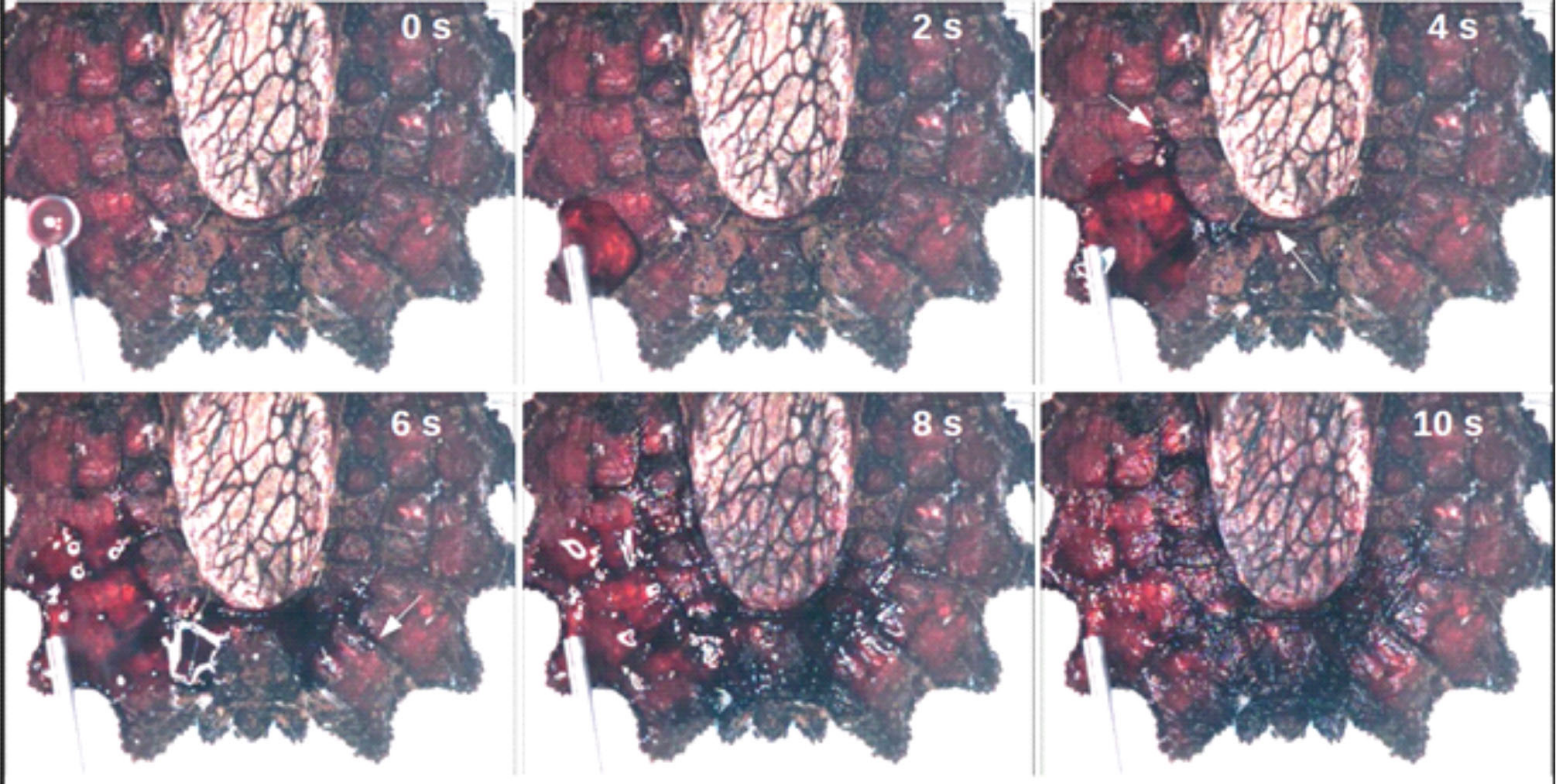
**Time course of wetting of *Dysodius magnus*.** A droplet of approximately 5 μl was applied onto the connexival plates. The water spread, finally covering the dorsal abdomen of the bug. Two effects can be observed: (1) the water spreads onto the surface of the conexival plates with local velocities of up to 0.1- 2 mm/s, and (2) the water enters the intersegmental sutures in between the connexival plates (indicated by arrows). In the channels the transport is much faster than the spreading on the surface. However, the water occasionally leaves the channels and spreads on the surface at the corresponding positions.

As shown in Fig. 1, *Dysodius magnus* changes its colour when wetted to adapt to the colour change of bark dependent on its humidity. About 5 to 10 µl of water is sufficient to completely wet the dorsal surface of a bug. For *D.* *lunatus* an identical behaviour could be observed (not shown). A detailed view on the time course of wetting is depicted in Fig. 2 and is shown in Movie 1. Within 10 s, a drop of 5 µl water spreads virtually all over the dorsal abdomen of the animal. The local velocities of the liquid front show a very high variability. One can measure from 0.1 up to about 2 mm s^−1^ dependent on the exact location where measured. As evident from Fig. 2 and from the corresponding video, the water spreads onto the surface of the connexival plates and the glabrous areas with rather low local velocities where the water enters capillary channels in between the connexival and glabrous areas (indicated by arrows in Fig. 2). In the open channels, the transport is obviously much faster than spreading on the surface. However, although faster transport can be achieved in the channel, it appears that it is energetically favourable for the water to be spread on the surface. As can be seen, the water occasionally leaves the channels and spreads (slowly) on the surface at the corresponding positions resulting in a thin water film covering the whole dorsal surface of the animal. This goes hand in hand with a change of the colour.

All these observations were made identically with *Dysodius lunatus*. Both animals show the same behaviour and the same morphology and chemistry (see below). Thus, for shortness and simplicity, only *Dysodius magnus* will be treated in detail in the following sections.

**Fig. 3.**
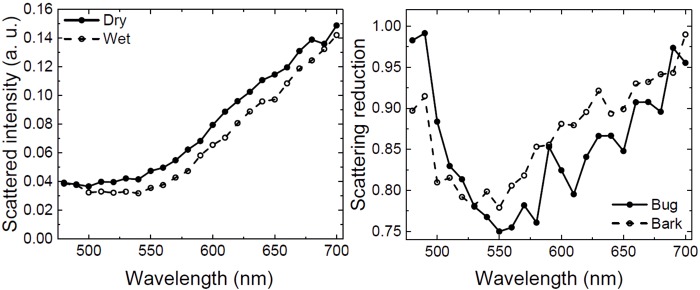
**Reflective behaviour of bug and bark.** (A) Reflectance (in arbitrary units, a.u.) of the surface of a dry and a wetted specimen of *Dysodius magnus*, measured in the integrated sphere versus the wavelength. (B) Relative scattering reduction (in percent) of bug and bark when comparing dry state versus wet state, plotted against the wavelength.

As already described, a wetting of the surface – be it bug or bark – will result in the change of optical properties (as shown in Fig. 1C-F). Measurements of the diffuse reflection of both surfaces verify this observation. Fig. 3A shows that, especially for wavelengths between 500 and 725 nm, a change in reflectance can be observed. From Fig. 3B one can see that the relative diffuse reflection reduction (=scattering reduction; i.e. the percentage of reduction when dividing dry=bright by dark=wet) of bark and bug is also following a very similar trend throughout the investigated spectra.

### Surface morphology

**Fig. 4.**
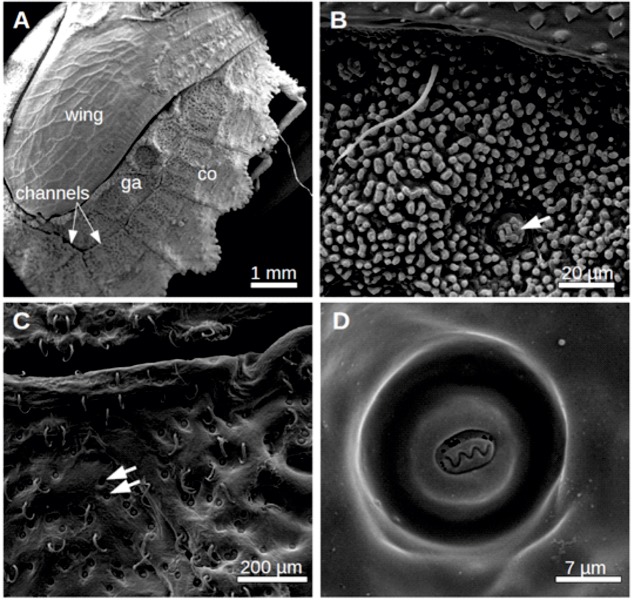
**SEM-images of the dorsal surface of *Dysodius magnus*.** (A) Overview of the dorsal abdomen. Already at this magnification a microstructure at the connexival plates (co) and the glabrous areas (ga) can be seen. Between the plates, intersegmental sutures occur. (B) Microstructures are naps of about 2 μm diameter, spaced by about 4 μm. In between circular structures can be seen (arrow). (C) After removal of cuticular waxes by hot 10% KOH the microstructures are gone and the circular structures (arrow) become clearly visible. (D) One circular structures at higher magnification can be seen.

To understand how the wetting can be achieved, the surface morphology of the flat bugs was investigated by scanning electron microscopy (SEM). A typical scan of the dorsal surface of *D.* *lunatus* is shown as an overview in Fig. 4A and in detail in Fig. 4B. Clearly, a microstructure covers the connexival plates as well as the glabrous areas. Furthermore, the sutures separating segmental borders are visible. These are here functioning like capillary channels. At higher magnifications one can see a rough surface, i.e. that the microstructures are small naps. These naps have a diameter of 1.96±0.37 µm and an average separation of 4.88±1.38 µm (*n*=100). The height of the naps can be measured to be in the range of 2.5 to 4 µm. In between the naps occasionally circular structures can be seen, such as the one marked by an arrow in Fig. 2B. These structures have typical diameters of 21±3 µm and are spaced by about 90±40 µm (*n*=100). The naps were assumed to be made of cuticular waxes and it was tempting to speculate that these wax structures are involved in the observed wetting behaviour, since they represent the surface coming in contact with water (even though waxes usually show a hydrophobic behaviour, an explanation for this was found later and is given in detail in the following chapters of this work). In order to test this, two bugs, which initially exhibited clear wetting (spreading of the water), were treated with hot 10% KOH solution for 60 s, to remove wax by means of hydrolysis leaving only the underlying chitin surface intact. After this treatment, the bugs were not wettable anymore but became water repellent (showing contact angles >90°). To give an estimation of the contact angle situation found on these bugs, refer to Fig. S4.

The surface morphology of these de-waxed animals is shown in Fig. 4C and D. Clearly the naps are removed. The circular structures, which are under native conditions covered with wax-naps at high density (Fig. 3B, indicated by the arrow), are again visible (indicated in Fig. 4C by arrows) but the wax is removed. In the higher magnification (Fig. 4D) clearly the naps are gone. It seems that the orifice of a secretion channel can be seen which exhibits a sinus shape. We assume at this stage, that these circular structures with the sinus-shaped openings in the centre are the orifices of wax glands, which produce the nap-shaped cuticular waxes.

Taken together we see that by KOH-treatment the waxy naps can be removed which concomitantly results in loss of the wetting capability of the cuticle. Thus, the waxy naps are vital for the wetting.

### Wax analysis

**Fig. 5.**
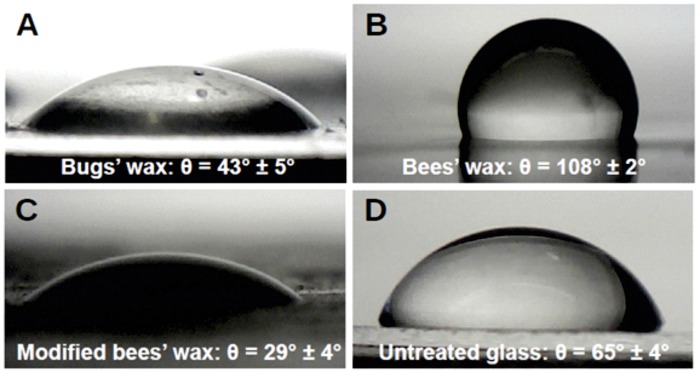
**Contact angle measurement of wax surfaces adsorbed on glass.** The removed and readsorbed wax from *Dysodius magnus* (A) exhibits a contact angle of 44° and is therefore hydrophilic, bees’ wax (B) clearly shows a hydrophobic behaviour with a contact angle of 108°. If however the bees’ wax is modified by adding eruccamide (C) or other fatty acids or soaps (not shown), the contact angle can be reduced again. In the example of eruccamide the contact angle dropped to 29°. For comparison the untreated glass (D) exhibits an contact angle of 65°.

In order to get a first idea of the physical and chemical properties of the cuticular wax, especially the outer layers which directly come into contact with water, the wax had to be removed gently from the cuticle without dissolving deeper layers or substances from within the bug's body. This was done by washing the animals with chloroform. Transmission electron micrographs of so treated animals show that in fact only the outer layers of the cuticular wax was removed, but basal wax was still present on the bug's body (shown in Fig. S3A and B). Notable at this point is that the removal of only these outermost waxes was enough to render the bugs hydrophobic. The chloroform containing the dissolved waxes was dripped onto glass cover slips and was then allowed to evaporate, resulting in a thin white film sustaining on the glass. SEM revealed that the surface of this wax film was virtually flat (not shown). When applying water onto the wax film, a surprisingly low contact angle of 43.9°±4.7° (*n*=15) was measured. A typical example of such a contact angle measurement is shown in Fig. 5A.

To determine which molecules are present in the bugs' waxes, they were dissolved and subjected to gas chromatography with subsequent mass spectrometry (GC-MS). This first analysis yielded some main substance classes that could be identified. Besides mono-, di- and triglycerides with chain-length ranging from C 12 to C 29, many free fatty acids (mono- and di-carboxylic acids) were detectable. We could not determine whether these were really fatty acids or soaps, i.e. salts of the fatty acid. An exact quantification of the amounts of the different substances was not possible due to the small amounts of samples available. Furthermore, high-performance liquid chromatography mass spectrometry (HPLC-MS) was performed in order to verify the GC-MS findings. Derivatization was not required in case of HPLC-MS. Detection was carried out in positive mode to be able to detect the glycerides. Glycerides were observed almost exclusively as sodiated [M+Na]^+^ pseudomolecular ions. Expected fatty acids were detected as protonated [M+H]^+^ as well as (in some cases more abundant) sodiated [M+Na]^+^ species. Saturated and unsaturated fatty acids (mono- and di-carboxylic acids) were detected. For some compounds, a characteristic water loss due to in-source fragmentation was observed indicating that hydroxylated fatty acids are also present in the sample. Overall, the complementary HPLC-MS measurements confirmed the previous GC-MS findings, but, in total, a smaller number of compounds was detected. Additionally to the fatty acids and glycerides, significant amounts of erucamide (cis-13-Docosenoamide) were identified in HPLC-MS. This substance is well known to be an often-found contamination in analytical chemistry. We are aware of this and therefore all treatment of the substances was done with glass equipment only, avoiding any lab plastics. All glassware was initially cleaned with chloroform (analytical grade) in order to avoid contaminations. Still, fatty acids, soaps, and the dubious erucamide, had at least one commonality: all these substances have polar and non-polar sides (amphiphilic character) and make therefore good candidates to act as mediators between hydrophobic insect wax on the one side, and polar water molecules on the other. Different researchers already showed that aliphatic molecules can be arranged perpendicularly orientated (with regard to their longitudinal axis) on cuticular waxes of plants (Domínguez et al., 2011; Graça, 2002). This gives a high possibility for amphiphilic substances to do the same on insect cuticle waxes.

To test whether the erucamide and/or the free fatty acids or soaps are able to render insects wax hydrophobic, bees' wax was modified. As can be seen in Fig. 5B, bees' wax is hydrophobic, exhibiting a contact angle of about 108°. If simply melting the bees' wax and applying soaps, fatty acids or erucamide at small amounts to the liquefied material, the contact angle could only be changed marginally by a few degrees (not shown). Only when adding strong detergents, like octoxinol-9, could the contact angle be lowered significantly. However, when an initial film of bees' wax was made and then soaps or erucamide was applied on top of the film as solution in chloroform or alcohol, a significant effect on the contact angle could be observed. Fig. 5C shows the effect of erucamid-application on a pre-established wax film. Here saturated erucamid-solution was applied and was allowed to dry on the wax. Then the surface was rinsed with water. Clearly the contact angle drops to very hydrophilic values of about 30°. However, in the SEM even the modified wax appears to be flat and no spontaneous nap-formation could be observed (not shown).

An exact quantification of the contents was not possible due to the layered structure resulting from the production of the modified wax layer. Nevertheless, it appears clear that the found substances from GC-MS can reduce the contact angle to values observed on natural flat bug's wax.

### Morphology of wax glands

**Fig. 6.**
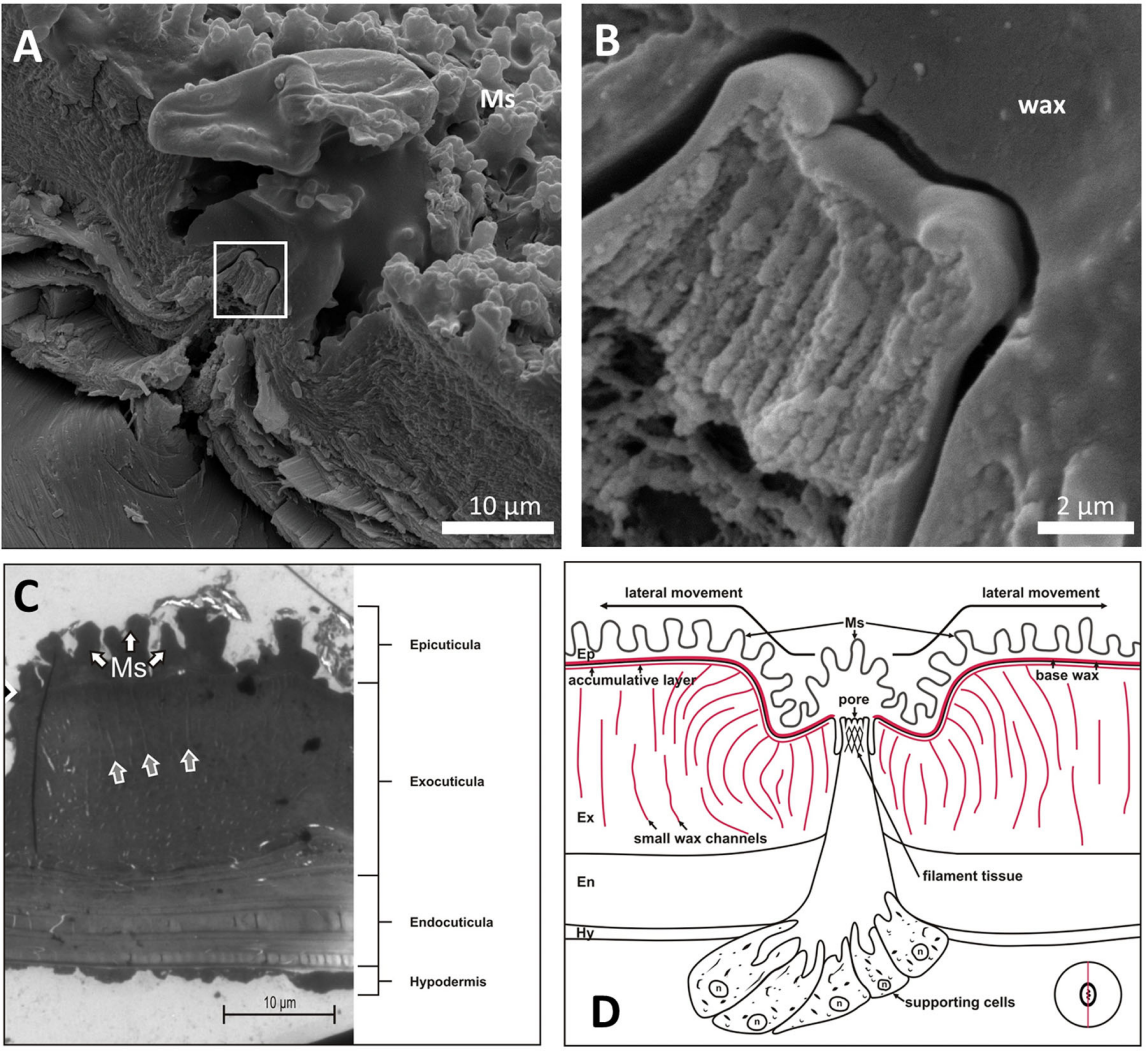
**Morphology of the wax secreting glands.** (A) Freeze fracture through one of the circular structures depicted in Fig. 4, which are presumably wax glands. In between the superficial microstructures (Ms) a rim with a filamentous tissue in the middle can be seen. (B) Higher magnification of the area marked with the rectangle in A. (C) TEM image of an ultra-thin section through the cuticula in the vicinity of a gland. A typical layered structure can be seen [white arrows showing the microstructure (Ms), also called naps]. In the exocuticula small channels can be seen (grey arrows). (D) Proposed morphology of the wax glands. The insert shows the cutting direction.

To understand if a layered structure of the cuticular waxes can be explained, morphological analyses of the presumable wax glands, i.e. of the circular structures observed in the SEM, were performed. Freeze fracture through one of the circular structures, which are presumably wax glands, can be seen in Fig. 6A and B. In between the superficial microstructures (Ms) a rim with a filamentous tissue in the middle can be seen. This becomes rather obvious when looking at higher magnification in Fig. 6B. Additionally TEM-images of an ultra-thin section through the cuticula in the vicinity of a gland were recorded. One typical image is shown in Fig. 6C where the typical layered structure of the cuticle can be seen. In the exocuticula small channels can be seen (arrows). Additional TEM-images of the cuticle are shown in Figs S1 and S2. Taking together the freeze fracture and the TEM-images, we propose a morphology of the wax glands as depicted in Fig. 6D as will be discussed below.

### Mimicking the wetting behaviour on polymer foils

**Fig. 7.**
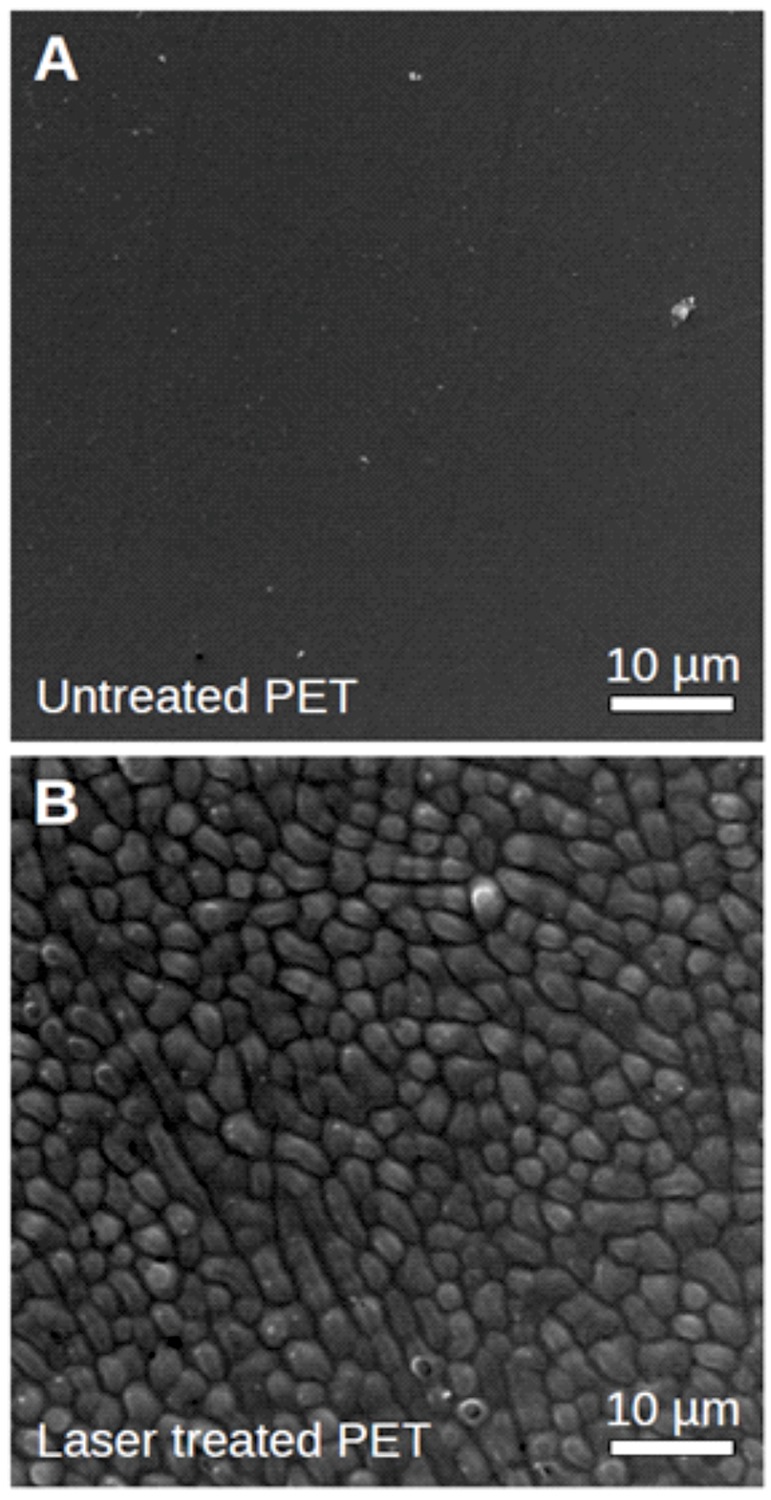
**Surface morphology of PET-foil.** The untreated foil (A) was found to be virtually flat. If the PET was treated by a pulsed UV laser (B), individual naps can be induced. These naps are with respect to the surface area amplification very similar to the microstructures observed on *Dysodius magnus* and *D. lunatus*, resulting in similar values for the Wenzel roughness (*r* is approx. 1.4 for the bugs and 1.2 for the PET foil and thus determining the needed contact angles for Cassie-Baxter impregnating with 50.5° and 48.2°, respectively).

In order to see if the wetting (water spreading) behaviour which can be seen on native *D. lunatus* can be fully explained by the findings so far, an attempt was made to mimic the effect. For this purpose, naps on PET-foils were produced by irradiation with nanosecond UV laser pulses. The spontaneous formation of the quasi-periodic naps is self-organised due to the release of the biaxial stress-fields and crazing in the irradiated but not ablated surface (Arenholz et al., 1991). Fig. 7 shows the surface morphology of PET-foil as seen in the SEM. While the untreated PET is virtually flat, areas which were irradiated with the laser are covered by naps. These naps have a diameter of about 2 μm. The PET-naps are slightly denser (average distance 3.5 µm) than the naps observed on *Dysodius*, but on the other hand they are not that high (about 1 µm) resulting in similar surface enlargement when compared to the natural archetype (see Discussion section).

**Fig. 8.**
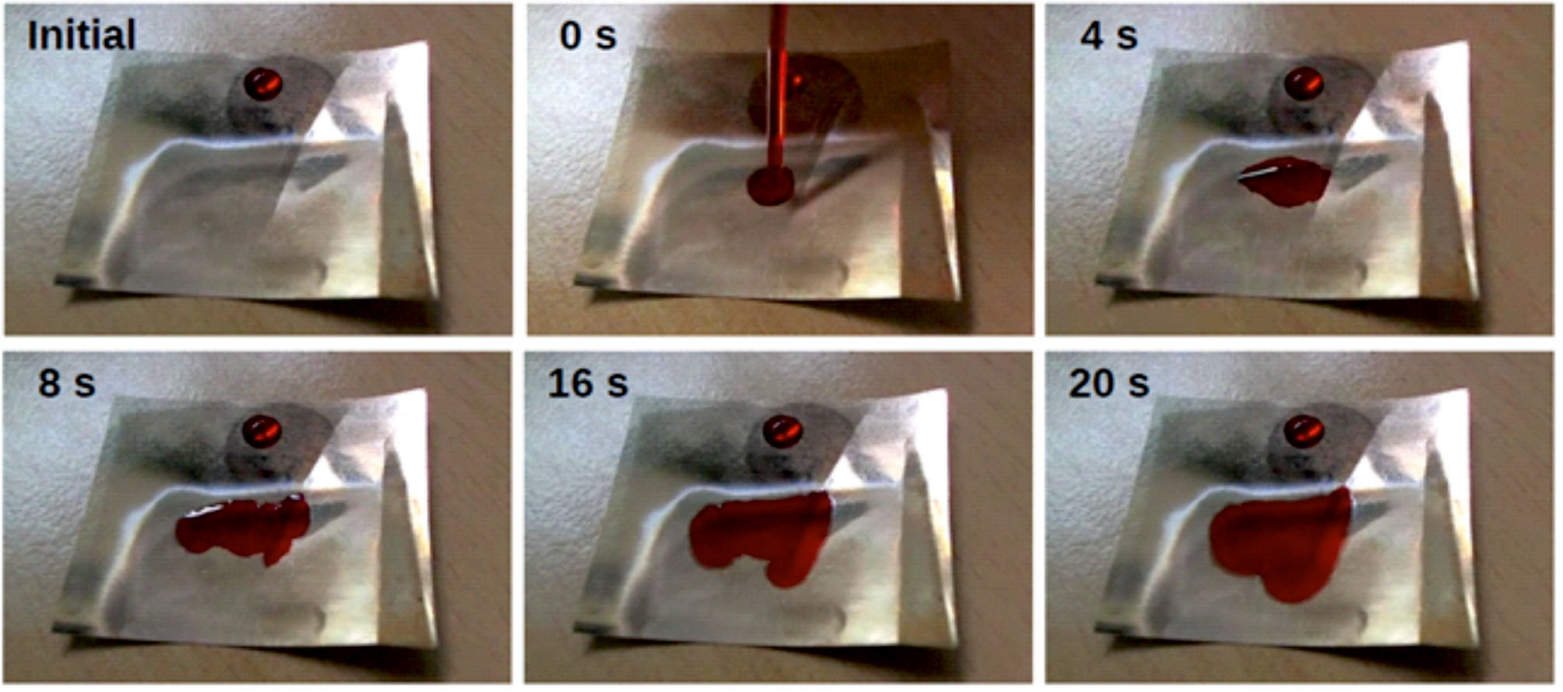
**Time series of the behavio**u**r of dyed demineralized water on a PET-foil mimicking the surface topography of *Dysodius lunatus* and *D. magnus*.** The PET was partially laser-structured. The glossy part (upper, left and right margins) is untreated, while the opaque part in the lower centre is structured by laser processing as shown in Fig. 7B. Initially a drop of dyed water was placed on the unstructured PET. Obviously the droplet sits stable with a contact angle of about 40° (± 3.5, *n*=10). If dyed water is applied onto the laser-structured part, the water immediately begins to spread with an apparent contact angle below 10°. At the waterfront, a margin with slightly different colour can be observed. We assume that here the water is in between the microstructures. The PET-foil piece has a width and length of 40 mm.

The native PET-foil is slightly hydrophobic. In order to mimic the wax material with an intrinsic contact angle (contact angle on the flat surface) of about 40°, we treated the foils with a sputter coater. Small amounts of gold were deposited in an argon plasma until a contact angle of 40° was reached (same coating procedure as stated for SEM: 180 s at 1 kV, resulting in a layer thickness of approximately 20 nm). The wetting behaviour of such foils is shown in Fig. 8. The glossy part (upper, left and right margins) of the foil shown there is not irradiated, corresponding to the flat foil shown in Fig. 7A. However, the opaque part in the lower centre of Fig. 8 is structured by laser processing as shown in Fig. 7B. Initially a drop of dyed water was placed on the unstructured PET. A stable droplet is formed which stays at a contact angle of about 40°. On the contrary, if water is applied onto the laser-structured part, the water immediately begins to spread and to form a film of water. The apparent contact angle is below 10°; thus the surface is superhydrophilic. At the water front, a margin with slightly different colour can be observed. Movie 2 shows this experiment.

## DISCUSSION

In the present study we found that the excellent wetting properties of *D.* *lunatus*, which are a prerequisite for its self-adaptive camouflage, are due to a micro-structured wax surface. The waxes are hydrophilic due to the superficial addition of amphiphilic molecules like soaps, fatty acids, monoglycerides or erucamide. It was shown that superficial addition of these substances to typical insects' waxes can render these waxes hydrophilic. Unfortunately, due to the very limited access to the native wax, we cannot give a quantitative analysis of the components and we cannot definitely show that some fatty acid anions appear as soaps.

As shown in the Supplementary material, only the superficial layers of the cuticular wax can be removed by chloroform-treatment while removal of the basal wax layers requires hot KOH. Thus, the chemical composition is not uniform. The morphology of the presumable wax glands found in *D.* *lunatus* fits to this observation: in the vicinity of the presumable wax glands, small channels through the exocuticle can be found, as they are also described in Lockey (1985) to be the insects’ main body-wax secretion instrument. These are assumed to support the stable but hydrophobic basal wax. The complexly built wax glands with the sinus-shaped secretion opening, on the other hand, appear to supply wax onto this basal wax layers. Blomquist and Jackson (1979) found that cuticle waxes of insects usually are compounds of longer chain hydrocarbons, which obviously need to be produced somewhere in the insects body. Foldi (1981) showed that insect wax glands exhibit so called support-cell structures often directly attached to channel systems of glands. As shown in the Supplementary material, we found such support cells for *Dysodius* glands too, and cell organelles like endoplasmic reticula, oenocytes and lamellar bodies were observed in high quantities. These are all structures, which usually are directly involved in a cells fat/lipid household, as shown by Lockey (1988) and Schmitz and Müller (1991). Additionally, somehow these glands manage to form the nap-structure when secreting the wax. This mechanism is not fully explainable so far, but it has been shown that similar complex glands are able to produce highly sophisticated wax structures for different insects, as also shown by Foldi (1981).

It is worth noting, that the nap structure is not self-assembled in any of our experiments. The removed and readsorbed native waxes as well as the artificially modified bees' waxes never spontaneously formed naps similar to those observed at the surface of the flat bugs under investigation.

How do the hydrophilic waxes with contact angles of about 40° in combination with the observed naps yield a superhydrophilic surface? Observing the natural archetype or the structured PET-foil, one can see that in fact a drop of water applied to these surfaces sits on a substrate while the liquid appears to penetrate the interconnected grooves in between the naps. Thus the droplet faces a patchwork of solid and liquid. This is exactly the scenario of the so called Cassie-Baxter impregnating wetting, also called hemi-wicking. This wetting has to be distinguished from the pure Wenzel wetting (Wenzel, 1936). In the latter case, the solid outside of the triple line is dry, whereas in the Cassie-Baxter impregnating situation the grooves, acting as capillaries, are wetted. The apparent contact angle θ*^C^* of the drop sitting atop of the structure is:(1)
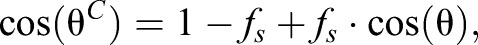
where θ is the contact angle of the unstructured solid material and *f_s_* is the relative fraction of the solid underneath the droplet. As explained in detail in Bormashenko (2013), Cassie-Baxter impregnating wetting is possible if and only if the contact angle of the unstructured material θ fulfils(2)
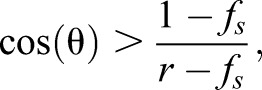
with *r* denoting the Wenzel-roughness. This is the ratio of the total surface area and the projected area, i.e. the factor of surface enlargement due to the structuring. If a regular array of cylindrical naps of radius *R* spaced by a distance *d* occurs, the ratio *f_s_* follows to be:(3)
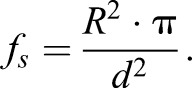
Thus, in our case of *Dysodius f_s_*≈0.3 and in the case of the naps on the PET-foil *f_s_*≈0.6, the Wenzel roughness can be estimated to be *r*≈1.4 in the case of the natural archetype while for the PET-foil one can estimate *r*≈1.2. This leads to the condition that for the *Dysodius* surface structure to allow Cassie-Baxter impregnating wetting the contact angle of the wax needs to be below 50.5°. For the artificial naps on the PET, the contact angle has to be below 48.2°. Not surprisingly this is found in the corresponding cases.

It appears clear that Cassie-Baxter impregnating wetting occurs and a continuous water film covers the bug's dorsal surface. In order to understand the darkening effect, one has to understand generally why most objects appear darker when wet. The amount of light seen from an illuminated and not self-lucent object is mainly dependent on its albedo, i.e. the reflectivity of the surface. This albedo depends on several aspects, the two main aspects being (Twomey et al., 1986): (i) the total reflection within a continuous water film, and (ii) a modified reflection coefficient. The first aspect was proposed by Ångström (1925). The surface roughness leads to diffuse reflection and is thus dependent on the angle for total internal reflection. A rough surface can be treated as array of randomly oriented small facets reflecting specularly. Assuming this rough surface is covered by a thin water layer, light incident onto this layer has a probability (1-*R*) of reaching the rough surface. Here *R* is the reflection coefficient of the liquid-air interface. A fraction named *a* is then absorbed by the rough surface. Of the light reflected back by the rough surface, a fraction *p* is then reflected back by the liquid-air interface so that it is once again incident on the rough surface. This process continues *ad infinitum*. Thus, the absorption probability of the water covered rough surface follows to be:(4)
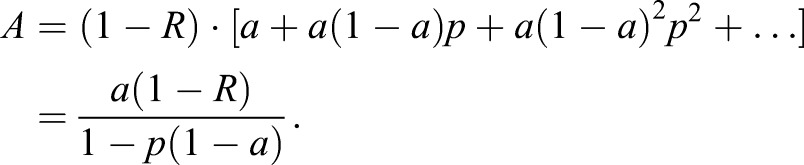
According to Ångström (1925) *p* can be evaluated for a Lambertian surface as the fraction of diffusely reflected light that lies outside a cone with critical angle q_C_, which follows to be:(5)
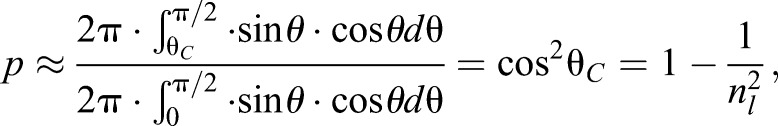
with *n*_l_ denoting the refractive index of the liquid. This is valid of course only for perpendicular illumination. If now the absorption *a* of the bug surface and of bark is identical and if both surfaces can be assumed to exhibit Lambertian (ideally matte) reflectance, the closed-water film will reveal an identical change of the albedo.

**Fig. 9.**
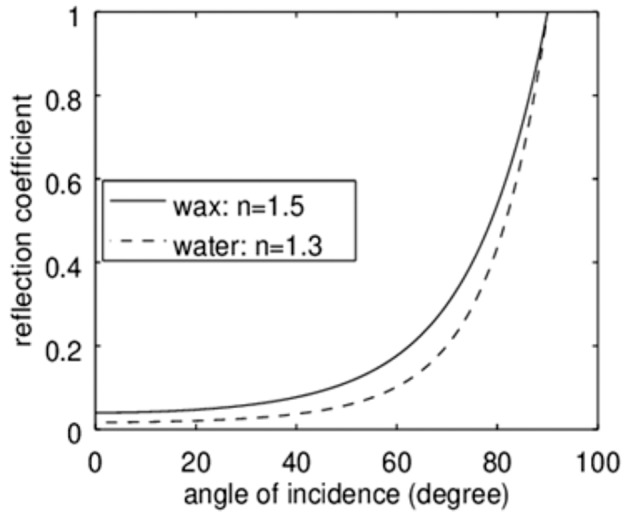
**Reflection coefficient dependent on the angle of incidence.** The functions were calculated according to eqn. 6, using values for bees’ wax and water covered bees’ wax as a model. Clearly, the wetted surface has a lower reflection coefficient for all angles of incidence and thus would appear darker.

If now the water film is not perfect and only a change due to a modified reflection coefficient can be observed, another theoretical consideration has to be made: at an interface of a medium 1 and a medium 2 (e.g. air–wax), the reflection coefficient *R* of an object illuminated under angle of incidence of α_1_ (denoted is air) is:(6)
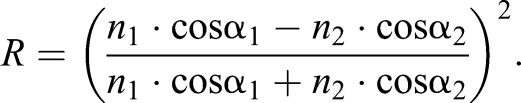
Here *n*_1_ and *n*_2_ denote the refractive indices of medium 1 and medium 2, respectively. The angle of incidence α_1_ and the angle of the refracted light α _2_ relate according to the refractive law as:(7)
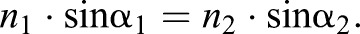
If medium 1 is air, then *n*_1_=1. When denoting the refractive index of medium 2 as *n* and the angle of incidence as α, we can simplify the equation for the reflection coefficient as:(8)
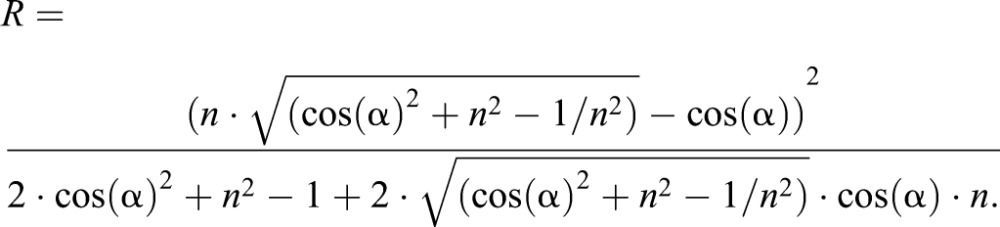
The refractive index of cellulose is approximately 1.5 (Fink, 1992), which is virtually the same as that of typical waxes like bee wax. The refractive index of water is 1.3. The behaviour of the reflection coefficient in dependence on the angle of incidence is depicted in Fig. 9 for *n*=1.5 (wax or cellulose) and for water. Clearly the reflection coefficient of the dry material is much higher than for water. Thus the dry material appears brighter while the wetted material, keeping a water film on the surface, is less reflective and thus appears darker. As the reflection coefficients of wax and cellulose are virtually the same, the degree of darkening is similar for wood and for the wetted bugs covered with a wax layer.

Of course, this is only a rough estimation neglecting further reflections on deeper layers, light scattering and the influence of other materials than wax and cellulose, which of course also appear in our samples. Furthermore *n*=1.5 is the refractive index of bees' wax. Due to the very limited amounts of bug's cuticular waxes, we were not able to measure the refractive index of this material. Nevertheless, it appears plausible that a matched change of the reflection coefficient of cuticular waxes and bark material is the clue for the adaptive camouflage exhibited by the flat bugs under investigation.

Finally, it remains to be determined why and how the water is transported through the capillary channels between the connexival plates and/or the glabrous areas and how it escapes from the channels to wet the surface. From the observation of the video (Movie 1) and image sequence (Fig. 2), it appears clear that slow imbibition wetting could occur all over the surface. However, the capillary transport is much faster yielding a complete wetting of the dorsal surface in short time even if only one droplet of water hits the animal at any position. To understand the dynamics of imbibition wetting, we follow the approach of Washburn (1921). At time *t* the imbibition length *l* of a liquid of surface tension γ and viscosity η from a reservoir into an array of randomly distributed micro-pillars of height δ and the roughness parameters ρ and φ as defined above follows to be:(9)
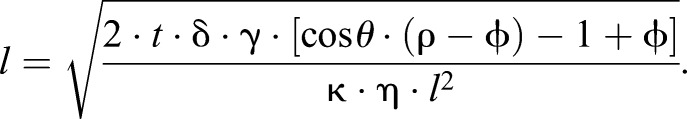
Here κ is a parameter defined as κ=1+ δ^2^·*p*^–2^·ln(*p*/σ), with *p* being the average centre-to-centre distance (pitch) of the pillars. This equation can be obtained by equating the viscous dissipation rate to the rate of change of the capillary energy (Chandra and Yang, 2011). Clearly a dense array, i.e. an array with small pitch *p*, leads to high capillary forces (wetting is energetically favourable). However, the viscous frictional losses due to the denominator in eqn. 9 (κηl^2^) slow the spreading, and the smaller *p* the slower the spreading.

On the other hand, in a relatively broad capillary channel although the driving capillary pressure is smaller than in between the pillars, the viscous friction is much lower. Thus, the water rapidly fills the network of capillary channels and from there the wetting of the surface occurs due to imbibition wetting. This can be shown easily as exemplified in Movie 3. Here a piece of glass was subjected to grinding so that the surface was rough and exhibited imbibition wetting. An open capillary channel of 500 µm width was then grounded into the glass using a diamond milling head. When applying a drop of water, one can clearly see that the channel is filled throughout its length (approx. 10 mm) rapidly and then from the filled channel the water starts the imbibition of the surface slowly but steadily. Thus the additional channels speed up the wetting process of the rather large *Dysodius* species.

Taken together, we can summarise that different bark bugs show the ability to change their apparent colour when wetted, matched to the colour change exhibited by tree bark when wetted. The wettability of the bugs is due to a rather hydrophilic wax on the cuticle which is structured by micropillars (naps) which allow imbibition wetting and will thus to form a stable water film. The formation of the film covering the whole dorsal surface is speeded up by capillary transport of the water through open capillary channels between the connexival plates and/or the glabrous areas. Here rapid filling of the whole network takes place due to the low viscous friction there. Then from the channels the slower imbibition wetting of the whole surface takes place.

## MATERIALS AND METHODS

### Video analysis

Videos for the investigation of liquid spread on the native surface of *Dysodius* were obtained with a Keyence highspeed video microscope (Model VW-9000; Keyence, Japan) with an attached 50× magnification lens (VHZ00R; Keyence, Japan). Videos were recorded at a frame rate of 125 fps.

Videos for the investigation of liquid spread on laser-generated structures on PET substrates were obtained with a digital camera Nikon D5300 (Nikon Corp., Japan) with an attached 60 mm macro lens (AF-S Micro Nikkor 1:2,8, Nikon Corp., Japan).

### Scanning and transmission electron microscopy (SEM and TEM)

SEM images were obtained with a Philips SEM 525 (Philips, Germany) at 15 kV. Air-dried bug samples were additionally dried on silica gel for 24 h before sputter coating with gold using a Polaron E5200 auto-coating device (former Polaron Unlimited, UK) at 1 kV for a duration of 180 s.

For TEM analysis, pieces of dried specimen were first fixated for 12 h at 4°C in 2.5% glutaraldehyde solution (SERVA, Germany) made from 0.2 M cacodylate buffer, followed by washing two times 15 min in pure cacodylate buffer. For further fixation and contrast enhancement, this was followed by 1 h incubation in 1% osmiumtetroxide (Fluka, USA) solution, again in cacodylate buffer, at 4°C; then two times washing for 15 min in pure cacodylate followed by two times 15 min in distilled water. Afterwards samples were dried in an ascending alcohol series (30%-70%). Negative staining was then applied by incubating in 1% uranylacetate (Serva, Germany, in 70% ethanol) for 2 h, before washing twice in 70% ethanol and finishing the drying row up to 100% ethanol. Afterwards ethanol was replaced by 2× 30 min in pure propyleneoxide (Serva, Germany) and followed by overnight incubation in a mix of two parts propylene oxide and one part epon (Epon quick mix, Serva, Germany). The samples were finally embedded in pure epon and polymerised for 72 h at 70°C.

Ultra-thin slices for TEM were cut with an ultra-microtome (OM 43, C Reichert, Austria) and diamond knives. After-staining of the slices was achieved by 0.2% lead citrate (Fluka, USA) and 0.2% uranyl acetate. The former incubated for 7 min, the latter for 20, followed again by washing of the samples after each step. For image acquisition, a Zeiss EM 10 (Zeiss, Germany) was used at 60 kV.

### Reflectance measurements

One dried specimen of *D. magnus* was mounted in an integrating sphere (15 cm diameter, Gamma Scientific, USA), normal to the incident laser beam. A supercontinuum source, i.e. a white light laser (NKT SuperK, NKT Photonics, Denmark) equipped with a Varia filter box, allowed colour tuning. The diffuse reflection of the bug is recorded via a custom photodiode facing the backside of the sample mount. The laser power was kept well below 1 mW to avoid optical and thermal damage. An overview of the setup used is given in the Fig. S4.

### Chemical analysis of cuticle waxes Part I: GC-MS

We used chloroform (Serva, Germany) to dissolve the topmost layers of cuticle waxes on one specimen of *D. lunatus*, as well as *D. magnus*, where we expected to find surface active components that interact with water. TEM images in the Supplementary material show that this attempt was successful (Fig. S3A,B). The samples' abdomens were carefully rinsed three to four times with 5 ml of chloroform, in order to dissolve surface components. Samples were then dried in a heating module (Reacti-Therm Heating Modul, PIERCE, USA), down to a volume of 50 µl, before derivatisation with 10 µl pyridine (Sigma-Aldrich, Germany) and 10 µl N,O-bis(trimethylsilyl)trifluoroacetamide (BSTFA, Sigma Aldrich, Germany) was done. Samples then were again dried at 70°C for 30 min before being re-dissolved in 50 µl chloroform and conveyed into auto-sampling veils. For *D. magnus* (first tested samples), this was the sole procedure. For gas chromatography, an Agilent 7890 A GC system (Agilent Technologies, USA) with mass spectrometer detector was used. A sample volume of 1 µl was injected via cool-on-column method into a DB1 column with a length of 30 m, 320 mm diameter and a stationary phase thickness of 100 nm. Carrier gas was helium, which was introduced for the first 5 min at 50 kP pressure before ascending to a final pressure of 150 kP in 3 kP/min steps. 150 kP were then held for 90 min. The heating protocol was 50°C at injection for two minutes, raised by 40°C min^−1^ until 110°C, held for two minutes at 110°C, raised by 3° min^−1^ until 320°C and then held for 50 min at this final temperature.

### Chemical analysis of cuticle waxes Part II: HPLC-MS

For HPLC-MS analysis the chloroform solution used to dissolve the topmost layers of cuticle waxes of two dried specimens of *D. magnus* was brought to dryness with a gentle stream of nitrogen in a sampling vial, and 0.5 ml acetonitrile was added to dissolve low molecular compounds. The resulting solution was used for injection. An Agilent 1100 series HPLC system (Waldbronn, Germany) equipped with a degasser, a quaternary pump and an autosampler was applied. Analytes were separated on a Kinetex C18 column (50 mm×4.6 mm, particle size 2.6 µm; Phenomenex, Aschaffenburg, Germany) using a water/acetonitrile gradient. Starting conditions were set to 75% solvent A (water with 0.1% formic acid) and 25% solvent B (acetonitrile with 0.1% formic acid). Mobile phase B was linearly increased to 90% within 19 min, which was then kept constant from minute 19 to 23. The gradient was changed back to starting conditions for re-equilibration; these conditions were held for 5 min. Flow rate was set to 1.0 ml min^−1^, temperature of the column heater was fixed at 30°C and an injection volume of 20 µl was selected. MS detection was carried out using an Agilent 6520 QTOF equipped with electrospray ionization (ESI) and operated in the positive mode. MS parameters were as follows: drying gas flow 10.5 l min^−1^, drying gas temperature 350°C, nebulizer pressure 50 psi, capillary voltage 3750 V, fragmentor voltage 175 V. The HPLC eluent flow was split (0.33 ml min^−1^) before entering the ESI source.

### Laser-generated structures on PET substrates

The experiments were performed on 50 µm thick flat, biaxially stretched Polyethylene terephthalate (PET) foils (Dupont, Mylar™). They were used without any pre-treatment.

Nap structures (small, pillar-like microstructures, which cover a surface) on PET foils were produced using the KrF excimer laser LPX 300 (Lambda Physik, Germany), operated at a wavelength of l=248 nm and a pulse length of about 20 ns. The repetition rate was set at 10 Hz. The naps were produced with typically 20 pulses using the full beam profile of the laser of a few cm^2^ at a distance of 25 cm from the laser output. The resulting laser fluence is 150 mJ/cm^2^ with some deviations due to intensity variations over the beam profile.

Test fluid for the PET foils was distilled water, dyed with 0.5% (w/w) Ponceau S (Carl-Roth GmbH+Co. Kg., Germany). Contact angles were measured with a droplet volume of 5 µl, using a custom made contact angle measurement setup.

## References

[BIO026070C1] Ångström A. (1925). The Albedo of various surfaces of ground. *Geogr. Ann.* 7, 323 10.2307/519495

[BIO026070C2] Arenholz E., Svorcik V., Kefer T., Heitz J. and Bäuerle D. (1991). Structure formation in UV-laser ablated poly-ethylene-terephthalate (PET). *Appl. Phys. A* 53, 330-331. 10.1007/BF00357196

[BIO026070C3] Blomquist G. and Jackson L. (1979). Chemistry and biochemistry of insect waxes. *Prog. Lipid Res.* 17, 319-345. 10.1016/0079-6832(79)90011-9 382185

[BIO026070C4] Bormashenko E. Y. (2013). *Wetting of Real Surfaces*. Berlin; New York: De Gruyter.

[BIO026070C5] Chandra D. and Yang S. (2011). Dynamics of a droplet imbibing on a rough surface. *Langmuir* 27, 13401-13405. 10.1021/la202208x 22004691

[BIO026070C6] Domínguez E., Heredia-Guerrero J. A. and Heredia A. (2011). The biophysical design of plant cuticles: an overview: research review. *New Phytol.* 189, 938-949. 10.1111/j.1469-8137.2010.03553.x 21374891

[BIO026070C7] Fink S. (1992). Transparent wood – a new approach in the functional study of wood structure. *Holzforschung* 46, 403-408. 10.1515/hfsg.1992.46.5.403

[BIO026070C8] Foldi I. (1981). Ultrastructure of the wax-gland system in subterranean scale insects (homoptera, coccoidea, margarodidae). *J. Morphol.* 168, 159-170. 10.1002/jmor.1051680205 30153717

[BIO026070C9] Graça J. (2002). Glycerol and glyceryl esters of ω-hydroxyacids in cutins. *Phytochemistry* 61, 205-215. 10.1016/S0031-9422(02)00212-1 12169316

[BIO026070C10] Heiss E. (1990). A review of the genus Dysodius Lepeletier & Serville 1828, with descriptions of two new species (Heteroptera: Aradidae). *Ann. Inst. Biol., U.N.A.M., Zool.* 61, 279-296.

[BIO026070C11] Holdgate M. W. (1955). The wetting of insect cuticles by water. *J. Exp. Biol.* 32, 591-617.

[BIO026070C12] Johansen A. I., Exnerová A., Hotová Svádová K., Štys P., Gamberale-Stille G. and Tullberg B. S. (2010). Adaptive change in protective coloration in adult striated shieldbugs Graphosoma lineatum (Heteroptera: Pentatomidae): test of detectability of two colour forms by avian predators. *Ecol. Entomol.* 35, 602-610. 10.1111/j.1365-2311.2010.01219.x

[BIO026070C13] Lockey K. H. (1985). Insect cuticular lipids. *Comp. Biochem. Physiol. B* 81, 263-273. 10.1016/0305-0491(85)90311-6

[BIO026070C14] Lockey K. H. (1988). Lipids of the insect cuticle: origin, composition and function. *Comp. Biochem. Physiol. B* 89, 595-645. 10.1016/0305-0491(88)90305-7

[BIO026070C15] Panizzi A. R. and Grazia J. (2015). *True Bugs (Heteroptera) of the Neotropics*. Dordrecht: Springer Netherlands Retrieved from http://ezproxy.unav.es:2048/login?url=http://link.springer.com/book/10.1007/978-94-017-9861-7.

[BIO026070C16] Schmitz G. and Müller G. (1991). Structure and function of lamellar bodies, lipid-protein complexes involved in storage and secretion of cellular lipids. *J. Lipid Res.* 32, 1539-1570. 1797938

[BIO026070C17] Schuh R. T. and Slater J. A. (1995). *True Bugs of the World (Hemiptera:Heteroptera): Classification and Natural History*. Ithaca: Comstock Pub. Associates.

[BIO026070C18] Silberglied R. and Aiello A. (1980). Camouflage by integumentary wetting in bark bugs. *Science* 207, 773-775. 10.1126/science.207.4432.773 17796010

[BIO026070C19] Twomey S. A., Bohren C. F. and Mergenthaler J. L. (1986). Reflectance and albedo differences between wet and dry surfaces. *Appl. Opt.* 25, 431 10.1364/AO.25.000431 18231193

[BIO026070C20] Washburn E. W. (1921). The dynamics of capillary flow. *Phys. Rev.* 17, 273-283. 10.1103/PhysRev.17.273

[BIO026070C21] Wenzel R. N. (1936). Resistance of solid surfaces to wetting by water. *Ind. Eng. Chem.* 28, 988-994. 10.1021/ie50320a024

